# Misconceptions about health and disease prevention behaviors of rural Appalachian Americans

**DOI:** 10.14419/ijm.v2i2.3572

**Published:** 2014-10-02

**Authors:** Wayne C. Miller, Brian N. Griffith, Timothy J. Bikman, Cameron M. Meyer

**Affiliations:** Center for Rural and Community Health West Virginia School of Osteopathic Medicine Lewisburg, West Virginia, USA

**Keywords:** Disease Prevention, Health Behaviors, Rural Appalachian Health, Self-Perceived Health

## Abstract

**Background:**

Appalachia is one of the unhealthiest regions in the United States due to poor disease prevention behaviors.

**Objective:**

Determine if self-perceived health of rural Appalachians is related to participation in disease prevention behaviors.

**Methods:**

Rural Appalachian adults (n=437) were surveyed regarding their self-perceived health and disease prevention behaviors. Healthy behaviors included: moderate (≥ 90 min/wk) and vigorous (≥ 45 min/wk) physical activity, sugared drink consumption (≤ 1 sugared drink/d), smoking (non-smoker), alcohol consumption (≤ 1 drink/d), blood pressure (≤ 120/80 mm Hg), and fast food consumption (≤ 1 time/wk). Participants were grouped where healthy = (self-health rating > 5 on a 0-10 scale), BMI < 25, and blood pressure (≤ 120/80 mm Hg). Jaccard Binary Similarity (JBS) coefficients and Russell and Rao (RR) dichotomy coefficients determined the association and predictability of self-health ratings and disease prevention behaviors. T-tests determined group differences in the number of disease prevention behaviors.

**Results:**

Individuals who reported being healthy had high JBS coefficients for having healthy sugared drink consumption (0.552), not smoking (0.704), low alcohol consumption (0.742), and low fast food consumption (0.481). RR results were similar to JBS results. Not smoking and low alcohol consumption were highly correlated (r=0.87). Those with a good health perception practiced more disease prevention behaviors (mean±SEM, 2.84±0.06) than those with a poor health perception (2.19±0.10, p<0.001). Good health perceptions were not strongly related to obesity and inactivity.

**Conclusions:**

Appalachians are not indifferent about their health. However, Appalachians may not understand how inactivity and obesity relate to disease.

## 1. Introduction

The formation of the United States Appalachian Regional Commission, in 1965, defined Appalachia as a specific geographical region consisting of 420 counties in 13 states, where 42% of the population is rural. Rural Appalachia is recognized as one of the least healthy regions in the United States. Realizing that many of the health concerns of rural Appalachia are modifiable, numerous health promotion programs have been instituted throughout Appalachia over the past two decades. Most of these programs and interventions targeting Appalachian health behaviors focus on diet, physical activity, smoking, and alcohol use. Undoubtedly, the intended outcome of these programs and interventions is a reduction in health disparities through individual behavior change.

An underlying theme in most of the major behavior change models is that a person needs to have a realistic perception of the behavioral issue (in this case an unhealthy behavior) in order to make a successful behavioral change (Baranowski et al. 2003). For example, the Knowledge-Attitude-Behavior model contends that some level of knowledge is prerequisite to the formation of healthier attitudes, and that the formation of new attitudes toward health will result in healthy behavior changes (Baranowski et al. 2003). The Behavior Learning Theory asserts that behavior is the outcome of a costs-to-benefits evaluation (Baranowski et al. 2003). Applying Behavior Learning Theory to eating behaviors means that a person will eat healthy if he/she perceives that the benefits of eating healthy outweigh the costs. Constructs of the Health Belief Model include a person's perceived susceptibility for contracting a disease, the perceived impact of the disease, the perceived benefits in taking action to reduce the threat of contracting the disease, perceived barriers to the preventive behavior, cues to action, and self-efficacy (Baranowski et al. 2003). The primary motivation to change behavior in the Health Belief Model is the magnitude of perceived threat of a disease or unhealthy condition. The primary motivational factor in the Social Cognitive Theory is also governed by perception, defined as the desire to achieve positive outcomes and avoid negative ones (Baranowski et al. 2003).

Perception is also the foundation of two of the most popular theories of behavior change – the Theory of Planned Behavior (TPB) and the Transtheoretical Model (commonly referred to as Stages of Change). The TPB argues that behavior is directed by one's attitude toward the behavior in question, the perceived social pressure to perform that behavior (subjective norm), and the ease with which one can perform the behavior (perceived behavioral control, Ajzen 1991). The combination of a positive attitude, a favorable subjective norm, and a high level of perceived behavioral control lead to a strong intention to perform the behavior. Similar to the TPB, perception is deeply rooted in the Transtheoretical Model (TM), only presented with different constructs. The TM proposes that a person makes a successful behavior change by progressing through certain stages of change (Prochaska et al. 1994). The first two stages, pre-contemplation and contemplation, rely totally on whether or not the person perceives that there is a problem (or need for behavioral change).

Thus, it seems like whichever philosophical model for health behavior one favors, the underlying theme for behavior change is individual perception; whether it be perception about the issue (attitude), perceived benefits/costs for performing the behavior, perceived risks of disease, perceived impact of disease, perceived social pressure to practice the health behavior (subjective norm), perceived control of the health behavior, or the perception of whether or not there is a health issue in the first place. One must recognize that the first step prior to behavior change in any theoretical model, then, is the perception that there is a problem. Using the TM as an example, if a person does not perceive health to be an issue (pre-contemplation and contemplation), then he/she will never progress through the stages of change in the TM. Similarly, if a person does not perceive health to be an issue, he/she will not acquire knowledge or attitudes to change behavior in the Knowledge-Attitude-Behavior model, evaluate costs and benefits of behavior change in the Behavior Learning Theory, form perceptions necessary for change in the HBM, develop perceived behavioral control in the TPB, etc.

Consequently, one's perception of health, or one's self-rated health, is a dominating factor that controls his/her health related behaviors. Since the health status of rural Appalachians has not improved much over the past two decades, in spite of numerous programs and interventions; it seems logical that rural Appalachians have not changed behaviors, because they perceive themselves as healthy. If Appalachians truly perceive themselves to be healthy, then interventions targeting health behavior change are going to continue to be ineffective. On the other hand, if Appalachians begin to perceive themselves as being unhealthy, then health behavior interventions are more likely to be successful.

The primary aim of this project, therefore, was to determine if self-perceived health of rural Appalachians is related to participation in disease prevention behaviors. The secondary aim of the project was to determine if healthy rural Appalachians practice more health promoting behaviors than unhealthy rural Appalachians. By accomplishing both of these aims, health care providers and public health professionals will be better able to motivate rural Appalachians to practice healthier behaviors to improve their health.

## 2. Methods

Rural Appalachian adults were surveyed regarding their self-perceived health and their disease prevention behaviors in order to determine the relationships among perceived health and disease prevention behaviors. Variables of interest were perceived health status, physical activity, sugared drink consumption, smoking, alcohol consumption, blood pressure, and fast food consumption. The study procedures were approved by the West Virginia School of Osteopathic Medicine Institutional Review Board for the Protection of Human Research Participants.

### 2.1. Sample population

The sample population consisted of 437 adults, aged 18-96 y, from all areas of rural Appalachia. The participants for the study were those who voluntarily submitted to a free behavioral health screening and blood pressure screening. The screenings were held in Lewisburg West Virginia, which is in the center of Appalachia. The entirety of West Virginia itself lies in the heart of Appalachia, a region characterized by poor health and health disparities.

### 2.2. Procedures

Participants in the study were given a survey containing 20 questions regarding gender, age, height, weight, smoking status, nutrition behaviors, exercise behaviors, blood pressure, use of medications, cholesterol levels, and self-rated health. Body weight and height were self-reported and were used to calculate body mass index (BMI). Self-reported height and weight have been shown to be valid and reliable (Spencer et al. 2002, Paradis et al. 2008). A classification of normal weight (NW), overweight (OW), and obese (OB) were assigned based on BMI stratification (NW ≥ 18.5 and < 25.0, OW ≥ 25.0 and < 30.0, OB ≥ 30.0). Smoking was evaluated by determining if the person was a non-smoker, current smoker, previous smoker; and how many cigarettes they currently smoke or previously smoked daily. Nutrition behaviors were assessed by two variables, sugared drink consumption and fast food consumption. A sugared drink, defined as regular soda, was understood to be a non-alcoholic sugar-sweetened carbonated drink. Regular sodas in the United States contain approximately 40 g of sugar and 150 kcal per 355 ml can. Drinking regular soda was dichotomized as a soda consumer (drink ≥ 355 ml soda per d) or non-consumer (drink < 355 ml soda per d, Rehm et al. 2008). Fast food consumption was dichotomized as fast food consumer (eating fast food 3 or more times a wk) and healthy food consumer (eating fast food < 3 times a wk, French et al. 2000). Exercise participation was dichotomized as sedentary (exercise ≤ 1 day per week for < 30 min) or active (exercise > 1 day per week for ≥ 30 min).

Blood pressure was measured manually, through auscultation. All blood pressure measurements were taken by a second year medical student, a staff nurse, or a phlebotomist from the West Virginia School of Osteopathic Medicine; who were under the supervision of a physician. Although only one blood pressure measurement is not suitable for a medical diagnosis, for the purpose of this study, hypertension was defined as having a systolic blood pressure ≥ 140 mm Hg or having a diastolic blood pressure ≥ 90 mm Hg. Use of medication was assessed by answering the question, “Do you take medication for any of the following: heart problem, blood pressure, diabetes or blood sugar problem, muscle or joint pain, cholesterol or blood fats, or depression”. Self-rated health was determined by ranking one's health on a linear scale. A self-rating of “healthy” was defined as a score greater than 5 on the scale of 0 to 10. The practice of six disease prevention behaviors were extracted from the questionnaire data: regular moderate physical activity, regular vigorous physical activity, healthy food intake, healthy sugared drink consumption, non-smoking, low alcohol consumption.

Each participant also provided their city, state of residence and/or zip code. Subsequently, the RUCA codes (Rural Urban Commuting Area codes) were used to verify that each participant came from a rural area in Appalachia. The RUCA codes correlate with postal service zip codes and classify United States census tracts using measures of urbanization, population density, and daily commutes.

### 2.3. Statistical analyses

All statistical analyses were performed using the Systat 12 statistical software package (Systat Software, Inc.; Chicago, IL, USA). Empty data cells were not included in the analyses. Group data are reported as mean ± SEM. Jaccard Binary Similarity (JBS) Coefficients were calculated to determine the proportion of times two events occur, given at least one event occurs. For example: if somebody reported being either healthy or obese, what was the chance that they reported being both healthy and obese? Russell and Rao dichotomy coefficients were also used to test the likelihood of two variables occurring simultaneously. T-tests were used to determine if those who perceived themselves as healthy practiced more disease prevention behaviors than those who perceived themselves as unhealthy. Statistical significance was declared at the P < 0.05 level.

## 3. Results

The demographics of the sample population are presented in Table 1. Using a survey size calculator (Raosoft 2014) it was determined that 385 participants were needed to get an accurate representative survey response of the Appalachian population, with a 95% confidence interval. A total of 437 rural Appalachian adults participated in the study. Therefore, the responses to the survey are representative of the rural adult Appalachian population.

Table 2 shows the proportion of respondents who considered themselves healthy according to self-perception of overall health, self-perception of weight health, and self-perception of blood pressure health.

Disease prevention behaviors are matched with health perceptions and presented in Table 3. Rural Appalachian adults who perceived their overall health and blood pressure health as good practiced more disease prevention behaviors than those with poor self-ratings for health. However, the number of disease prevention behaviors practiced did not differ for those with either a poor or good self-rating for body weight.

The JBS coefficients for the pairing of perceived health with disease prevention behaviors are shown in Table 4. The data reveal a very high likelihood of a respondent reporting being healthy overall, when he/she did not smoke and consumed low amounts of alcohol. Furthermore, there was a high correlation (r = 0.87) between smoking behaviors and alcohol consumption behaviors. There was a high likelihood that a respondent reported being healthy overall when he/she ate fast food infrequently and drank sugared drinks infrequently. On the other hand, people who thought they were healthy overall were not very likely to participate in regular physical activity. Results from the Russell and Rao dichotomy analyses revealed similar coefficients and findings to those seen with the JBS analyses (data not shown).

## 4. Discussion

The purpose of this project was to determine if self-perceived health of rural Appalachian adults is related to their participation in disease prevention behaviors. First of all, it is of interest that 74% of the Appalachians surveyed believed they were in good health. This finding is contradictory to what is known about Appalachian health. It is well established that the Appalachian region is one of the unhealthiest regions in the country (United Health Foundation 2014, State Data Center 2014). This disconnect between perceived health and morbidity is generally not seen in other populations (Rohrer et al. 2000, Ahmad et al. 2005, Thommasen et al 2005, Rohrer et al. 2007, Haseli-Mashhadi et al. 2009, Rohrer et al. 2010). Most often, there is a high correlation between self-rated health and morbidity, to the extent that the risk of mortality is sometimes more strongly associated with self-rated health than with objective health status (Mossey and Shapiro 1982). Further evidence of this disconnect between self-rated health and true health status in Appalachia can be drawn from some of the demographic questions in the current survey. The average BMI for the participants was 30.8, an obese classification. Only 21% of the participants were performing regular vigorous physical activity and just 32% performing regular moderate physical activity. A high percentage of the participants were on medications for chronic disease. Fifty seven percent of the participants were on medication for blood pressure, and 81% were on medicine for at least one of the following: heart disease, blood pressure, diabetes or glucose control, muscle or joint pain, cholesterol, or depression. Lastly, 92% of the participants said their doctor told them they had one or more of the following: heart disease, high blood pressure, diabetes or poor glucose control, muscle or joint problems, high cholesterol, lung disease, cancer, osteoporosis, or depression. All of these demographic data indicate that these Appalachian adults were a diseased population that thought they were healthy.

Respondents reporting being in good overall health and having good blood pressure health participated in more disease prevention behaviors than respondents reporting poor health for these two variables. These respondents (or many of them) would be the same ones that reported being on disease medications or having been told they have a disease. Whether they are truly healthy or not, the fact that they are practicing disease prevention behaviors demonstrates that they understand the connection between good health behaviors and blood pressure control and overall good health. On the other hand, the connection between body weight and health behaviors was not apparent in these Appalachians, since there was no difference in disease-prevention behaviors between those who perceived their body weight poor and those that perceived it as good.

One must not make the mistake of interpreting JBS coefficients the same as he/she would interpret simple correlation coefficients. If one were to erroneously compare the JBS coefficients to simple correlation coefficients, the interpretation of the JBS data would not seem as significant as they are. Since the JBS coefficient gives the proportion of times both events occur, given at least one occurs; an example of the correct interpretation is as follows: if somebody reported either being in good health overall or not smoking, the chance of them reporting that they were both a non-smoker and in good health overall is 74% (Table 4). The interpretation of these JBS coefficients becomes meaningful in that Appalachian adults see alcohol consumption and smoking as being very strong determinants of health. They see fast food consumption and sugared drinks as fairly strong determinants of health. However, they do not see physical activity as a significant determinant of health (Table 4).

The conclusive analysis is that Appalachians do not always properly associate their health status with their behaviors. The impact of the dissociation between self-rated health status and poor health behaviors in Appalachia is also demographically apparent. For example, more than 70% of Appalachia has the highest prevalence of obesity in the United States, with areas of Kentucky, Tennessee, and West Virginia having 80% of their population with obesity or its common co-morbidity, type 2 diabetes (Centers for Disease Control 2009). Despite state and federal policies targeting this epidemic, the problems of obesity and diabetes continue to plague the region, with few signs of reducing this trend. Results from this study suggest that one reason for limited success in fighting health disparities in Appalachia is based on distorted perceptions of health and disease-prevention behaviors. These data reveal that a high percentage (71%) of people who were classified as overweight or obese identified themselves as in good overall health. Also, about half of the people eating too much fast food (47%), drinking too many sugared drinks (47%) and who are sedentary (52%) identified themselves as healthy.

From the standpoint of behaviors leading to health risks, the results from this study may fall in line with what Manderbacka et al. (1999) discovered several years ago. They found no association between dietary fat avoidance and self-related health in Swedish adults; a finding that would parallel the Appalachians' dissociation between physical activity and self-rated health. The authors concluded that health behaviors are not considered directly when assessing one's own health, but the potential effect of behavior on self-rated health is mainly mediated by more specific health problems and their functional consequences. This may be the case with rural Appalachians. Physical activity may not be considered a health issue in Appalachia until functional limitations occur. Thus, poor health behaviors, such as inactivity, may not be associated with poor health until a direct personal link between those behaviors and functional limitations has been established in the mind of the individual.

Another possible explanation for the discrepancies between health perceptions and behaviors in Appalachia is the “common state of affairs”. It is very likely that the participants in this study came from families and communities in which the “common state of affairs” is to be unhealthy, and where disease-prevention behaviors are not practiced. Consequently, participants responded to the survey about their own health in this community context. Compared to the people with whom they are familiar (e.g., their family members, co-workers, neighbors, and community members) they may see themselves as healthy.

## 5. Conclusions

It appears that interventions focused on improving health behaviors in rural Appalachia are not going to be successful until the rural Appalachian's health perception becomes congruent with his/her health risk behaviors. High priorities for programs targeting health disparities in rural Appalachia should be those that target one's perception of health and/or one's perception of health risk behaviors. Moreover, public health programs targeting positive health behaviors may be more readily received than those targeting negative behaviors (Dave et al. 2009, Byun et al. 2010). For example, public messages about quick and easy ways to incorporate physical activity into one's current lifestyle may be received better than messages attacking the health risks of a sedentary lifestyle. Messages about how a healthy body weight improves functional capacity might be better received than messages about how obesity leads to sickness and early mortality. It should also be remembered that the formation and delivery of new public health messages and programs for rural Appalachians should be focused on people who are unhealthy and have poor health behaviors, but believe they are healthy. These messages and programs can easily be integrated into any one of the popular behavior change models. For example, this would mean focusing on moving people through the pre-contemplation and contemplation stage in the TM, focusing on the shaping of positive attitudes in the Knowledge-Attitudes-Behavior model and the TPB model, increasing benefits-to-cost ratios in the Behavior Learning Theory and HBM, increasing positive outcomes in the Social Cognitive Theory, and increasing perceived behavioral control in the TPB – schemas which all refer back to individual perceptions. The challenge, then, becomes formulating messages and programs about health and health needs which take into account the current distortion about health in Appalachia, the cultural context in which this distortion was shaped and recognized behavior theory.

## Figures and Tables

**Table 1:** Participant Demographics

Age	53 ± 1
Height (cm)	169 ± 1
Weight (kg)	84.2 ± 0.9
Body Mass Index (BMI)	30.8 ± 0.6
Men	192 (44%)
Women	245 (56%)

Values are Means ± SEM.

**Table 2:** Self-Perceived Health Status

Health Perception	Poor Rating	Good Rating
Healthy Overall	114 (26%)	323 (74%)
Healthy Body Weight	245 (56%)	192 (44%)
Healthy Blood Pressure	162 (37%)	275 (63%)

**Table 3:** Number of Disease Prevention Behaviors Practiced

Health Perception	Poor Rating	Good Rating	Significance
Healthy Overall	2.19 ± 0.01	2.84 ± 0.06	p < 0.001
Healthy Body Weight	2.80 ± 0.11	2.62 ± 0.06	p = 0.143
Healthy Blood Pressure	2.45 ± 0.10	2.80 ± 0.07	p < 0.002

Values are Means ± SEM.

**Table 4:** Jaccard Binary Similarity Coefficients for Good Health Overall Perception and Disease Prevention Behaviors

Good Health Overall Perception	
Good Health Overall Perception	1.000
Vigorous Physical Activity	0.256
Moderate Physical Activity	0.321
Healthy Fast Food Consumption	0.481
Healthy Sugared Drink Consumption	0.552
Not Smoking	0.704
Low Alcohol Consumption	0.742
